# Unsupervised learning for real-time and continuous gait phase detection

**DOI:** 10.1371/journal.pone.0312761

**Published:** 2024-11-01

**Authors:** Dollaporn Anopas, Yodchanan Wongsawat, Jetsada Arnin

**Affiliations:** 1 Biodesign Innovation Center, Department of Parasitology, Faculty of Medicine Siriraj Hospital, Mahidol University, Bangkok, Thailand; 2 Siriraj Integrative Center for Neglected Parasitic Diseases, Department of Parasitology, Faculty of Medicine Siriraj Hospital, Mahidol University, Bangkok, Thailand; 3 Department of Biomedical Engineering, Faculty of Engineering, Mahidol University, Bangkok, Thailand; West Virginia University, UNITED STATES OF AMERICA

## Abstract

Individuals with lower limb impairment after a stroke or spinal cord injury require rehabilitation, but traditional methods can be challenging for both patients and therapists. Robotic systems have been developed to help; however, they currently cannot detect the continuous gait phase in real time, hindering their effectiveness. To address this limitation, researchers have attempted to develop gait phase detection in general using fuzzy logic algorithms and neural networks. However, there is a paucity of research on real-time and continuous gait phase detection. In light of this gap, we propose an unsupervised learning method for real-time and continuous gait phase detection. This method employs windows of real-time trajectories and a pre-trained model, utilizing trajectories from treadmill walking data, to detect the real-time and continuous gait phase of human on overground locomotion. The neural network model that we have developed exhibits an average time error of less than 11.51 ms across all walking conditions, indicating its suitability for real-time applications. Specifically, the average time error during overground walking at different speeds is 11.20 ms, which is comparatively lower than the average time error observed during treadmill walking, where it is 12.42 ms. By utilizing this method, we can predict the real-time phase using a pre-trained model from treadmill walking data collected with a full motion capture system, which can be performed in a laboratory setting, thereby eliminating the need for overground walking data, which can be more challenging to obtain due to the complexity of the setting.

## Introduction

In the pursuit of advancing rehabilitative outcomes in healthcare for patients impacted by strokes and spinal cord injuries, it is pivotal to implement efficacious intervention strategies. While conventional physiotherapeutic modalities remain a cornerstone, there is a burgeoning interest in the integration of cutting-edge robotic systems in rehabilitation paradigms. These robotic systems play a vital role in alleviating the workload of physical therapists, while providing precise and consistent control over the rehabilitation protocols. Consequently, numerous studies are actively engaged in the development of such rehabilitation robotic systems. Recent progress in this field has led to the creation of advanced systems dedicated to gait training and assessment [1]. However, a notable limitation of current systems is their challenge in accurately controlling real-time gait for patients [2]. This limitation stems from the absence of systems capable of effectively detecting the patient’s gait phase in real-time. Gait phase detection holds significant importance in rehabilitation robotic systems, enabling precise and timely control of the patient’s walking patterns. Notably, research in this field has also focused on synchronizing the walking rhythm of the lower limbs with the upper limbs. This approach mimics natural walking patterns by utilizing rhythmic patterns of muscle activity that originate from thoracic and lumbar central pattern generators [3–6].

Numerous researchers have developed algorithms for gait phase detection using sensors or motion capture systems. For example, Skelly et al. utilized a force-sensitive resistor (FSR) sensor in conjunction with a fuzzy logic algorithm [7], while Hu and colleagues employed force-sensitive film paired with a customized algorithm for precise detection of gait events and phases [8]. Zhao and colleagues employed a pair of soft bend sensors affixed around the hip joint, complemented by a collection of flexible pressure sensors positioned on the plantar surface of the foot. These sensors were integrated into an adaptive gait phase detection system [9]. Similarly, Kong et al. and Bae et al. utilized an air tube and air pressure sensor with a fuzzy theory algorithm [10, 11]. Pappas et al. utilized a combination of FSR sensors and gyro sensors [12, 13]. Lim et al. developed and implemented a real-time gait phase detection algorithm using a ground reaction force measurement sensor and a motion intent sensor [2]. Tomc and his colleague employed a singular head-mounted Inertial Measurement Unit (IMU) in conjunction with adaptive frequency oscillators to accurately detect gait events [14].

Despite their strengths, many existing algorithms are limited by their focus on detecting discrete gait events, such as heel strike or toe-off, which only provide partial information about the gait cycle. This emphasis on contact detection, while useful in some scenarios, falls short in capturing the continuous nature of gait—a crucial aspect for dynamic and adaptive control [15–17]. Continuous gait phase detection, on the other hand, facilitates smoother transitions between phases and is essential for real-time applications such as rehabilitation devices and prosthetics, where achieving near-natural walking and effective posture correction is vital for enhancing patients’ quality of life [18]. Building on this need for continuous gait phase detection, our previous research utilized a motion capture system to analyze the trajectories of rats’ walking data and employed unsupervised learning algorithms to detect these continuous phases. This approach demonstrates significant potential for applying continuous gait phase detection in humans as well [19–21].

Additionally, it is important to note that phase synchronization between arms and legs is crucial for the recovery of movement function in stroke and spinal cord injury patients. Studies have shown that phase synchronization of upper and lower limb movement can lead to improved gait patterns and faster recovery of movement function [22–24]. By incorporating phase synchronization into rehabilitation robotic systems, patients may be able to recover their movement function more efficiently.

Mechanical sensors offer real-time gait analysis, allowing for the immediate identification of abnormalities and feedback during rehabilitation. In the field of gait analysis, common examples of mechanical sensors used include force sensors [25], inertial measurement units (IMUs) [26], and pressure sensors [27]. They possess numerous advantages such as non-invasiveness, portability, cost-effectiveness, and the provision of high temporal resolution, ease of use, and objective data, facilitating accurate and consistent gait analysis [28–30]. However, sensor-based studies can be limited by various factors such as accuracy, spatial resolution, environmental factors, sensor placement, durability, battery life, and data processing [31, 32]. IMU sensors, in particular, measure acceleration and rotational rates to capture detailed gait data. Unlike general mechanical sensors, which encompass a variety of devices such as force or displacement sensors, IMU sensors offer precise and comprehensive motion data. In addition to mechanical sensors, gait analysis can also make use of optical sensors, such as those found in motion capture systems [33]. These systems utilize multiple cameras for inspections and offer several advantages including high spatial and temporal resolution, accurate 3D analysis, objectivity, repeatability, versatility, and the ability to handle large data sets [34, 35], but are costly, space-intensive and require trained personnel [36]. Collecting overground walking data via a motion capture system necessitates a considerable amount of space, making the acquisition of large training data sets from overground walking data challenging.

In order to map the phases of the upper and lower limbs using signals from a motion capture system, a phase synchronization algorithm is necessary. There are several existing algorithms that can fulfill this role, including the Koopman operator theory, Poincaré maps, Hilbert-Huang transform, and continuous wavelet transform. These algorithms offer various advantages and limitations for gait analysis as shown in Table 1.

**Table 1:** Comparison of existing methods to map the phases of the upper and lower limbs.

Method	Real-time application	Continuous gait phases detection	Stability	Reliability	Complexity
Koopman Operator Theory [37, 38]	Yes	Yes	Stable as a linear representation. Outcome influenced by choice of observables; applicable to constant-velocity gait.	Reliable for understanding nonlinear dynamics, but dependent on the choice of observables and sampling.	Mathematically and computationally complex, especially for high-dimensional systems.
Poincaré Maps [39]	Yes	No	Depends on data periodicity. Not ideal for noisy or non-periodic data.	Reliable for periodic data to visualize phase space and attractors.	Graphical representation requiring some preprocessing for useful visualization.
Hilbert Transform [40]	No	Yes	Sensitive to noise; requires quasi-monotonic signal.	Reliable for extracting instantaneous phase and amplitude if data meets conditions.	Involves complex analytics. Requires preprocessing, e.g., filtering.
Continuous Wavelet Transform (CWT) [41]	No	Yes	Can handle non-stationary data due to time-frequency localization.	Very reliable with the right choice of mother wavelet and scales. Offers time-frequency representation.	Computationally intensive, especially for lengthy datasets.
Our proposed algorithm	Yes	Yes	Stable during real-time applications across different walking conditions.	Highly reliable for real-time gait phase detection across various walking conditions.	Employs a pre-training model from treadmill data, making it less complex than requiring overground walking data in a complex setting.

The Koopman operator theory is a data-driven method that extracts nonlinear dynamics from data and is computationally efficient, although it does require a significant amount of data [37, 38]. Poincaré maps are relatively simple and interpretable, capable of extracting key features of gait dynamics, but necessitate a priori knowledge of the gait cycle [39]. The Hilbert-Huang transform is robust to noise and can extract nonstationary and nonlinear features from data, but it also requires a priori knowledge of the gait cycle [40]. Finally, the continuous wavelet transform is versatile in handling different types of signals and can analyze nonstationary and transient features of data, but it is computationally complex [41].

Despite their strengths, these algorithms are still unable to perform effectively in real-time applications due to their massively computational requirements. To address this challenge, this study proposes an unsupervised learning approach to detect the continuous gait phase of humans in real time. This approach can detect gait phases across a range of speeds, as demonstrated by its ability to detect ongoing gait phase at different speeds of rats’ walking patterns in our previous study [19–21]. By utilizing training data obtained from treadmill walking, this algorithm is expected to detect the real-time continuous gait phase of overground walking data. Furthermore, this approach facilitates gathering a substantial quantity of walking data in a laboratory setting, which is less cumbersome than the collecting data from overground walking.

If successful, this algorithm is expected to enhance the design of rehabilitation robotic systems that control lower limb locomotion by determining ankle position, enabling patients suffering from stroke and spinal cord injury to attain a more natural gait pattern and potentially facilitate faster recovery of their walking abilities. Further studies are required to examine whether the benefits of transferring a pre-trained model from a controlled treadmill setting to infer untrained overground walking data in patients are sufficiently robust. Should the results demonstrate adequate performance, this method could effectively address the significant challenge of data collection, a critical component of the system.

## Materials and methods

### Dataset and selection criteria

Our study employed the open dataset presented by Fukuchi et al. in their 2018 publication [42] to train an unsupervised learning method for real-time detection of the continuous gait phase. The dataset comprises information on treadmill walking at various speeds, with each participant undertaking 90-second walks at each of the eight gait-speed conditions (40%, 55%, 70%, 85%, 100%, 115%, 130%, and 145% of their self-selected dimensionless speed in a randomized order), as well as overground walking at slow, comfortable, and fast speeds from a total of 42 individuals. The data was captured via a motion capture system operating at a sampling frequency of 150 Hz and was employed as the input for our neural network. The motion capture system employed in this study was a 12-camera Raptor-4 system (Motion Analysis Corporation, Santa Rosa, CA, USA) [42]. The training and testing data were selectively chosen solely from the range comprising thirty consecutive samples, as this criterion guarantees the manifestation of continuous normal walking. Consequently, gait data from two subjects were omitted due to the above reason.

**Fig 1:**
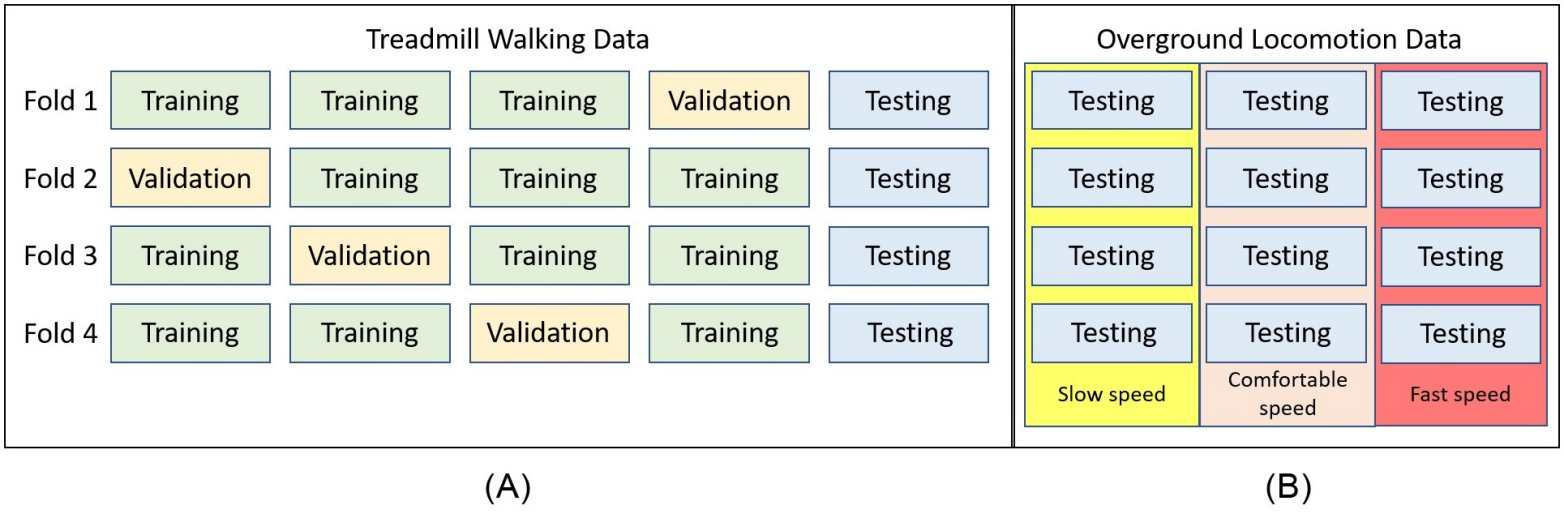
This study implements a 4-fold cross-validation and inferencing procedure. The dataset comprises (A) treadmill walking and (B) overground locomotion data, both divided into 4 folds. Each fold contains a training dataset from 24 subjects, a validation dataset from 8 subjects, and a testing dataset from 8 subjects for the treadmill walking data. Furthermore, the testing datasets for the overground locomotion data include three different speeds: slow, comfortable, and fast.

To ensure a fair evaluation, the data were randomly divided into three groups for training (N = 24), validation (N = 8), and testing (N = 8), in a ratio of 3:1:1 for each group as shown in Fig 1. Each dataset included a comprehensive range of relative speeds, from slow to fast walking, to ensure the algorithm accurately infers the various gait dynamics at different speeds. Additionally, a 4-fold cross-validation method was employed to test the testing dataset using all folds. After that, it was then utilized to test the testing data from overground walking data at three different speeds, as illustrated in Fig 1.

### Data preparation

Initially, the relative positions of x- and y- axes of human’s ankle are calculated by the following equations

$$\begin{array}{c} R_{x}=x_{Ankle}-x_{Iliaccrest} \end{array}$$ (1)

$$\begin{array}{c} R_{y}=y_{Ankle}. \end{array}$$ (2)

To determine the relative position of the x-axis of the human ankle, denoted as *R*_*x*_, and the position along the x-axis of the human ankle, denoted as *x*_*Ankle*_, a series of measurements is conducted. Similarly, the position of the x-axis of the human iliac crest, denoted as *x*_*Iliaccrest*_, and the relative position of the y-axis of the human ankle, denoted as *R*_*y*_, and the position along the y-axis of the human ankle, denoted as *y*_*Ankle*_, are also determined through a series of measurements. The positions of the iliac crest and ankle were identified using anatomical landmarks, as measured by Fukuchi et al [42]. This methodology ensured precise and consistent measurement of the gait parameters. Subsequently, the directional headings of the relative positions of the x- and y-axes are calculated by applying a computation on two consecutive windows of current and previous data. Each window contains seven neighboring samples. To obtain the first batch of data, 13 samples are required, spanning from the 1st to the 13th sample. With a sampling rate of 150 Hz, this initial data collection takes approximately 86.7 milliseconds (ms). However, for subsequent batches, overlapping windows are used. For example, the next batch of data will cover samples from the 2nd to the 14th sample, and then from the 3rd to the 15th sample, and so on. This overlap means there is no need to wait an additional 86.7 ms for each new batch of data, allowing for continuous and efficient data processing.

**Fig 2:**
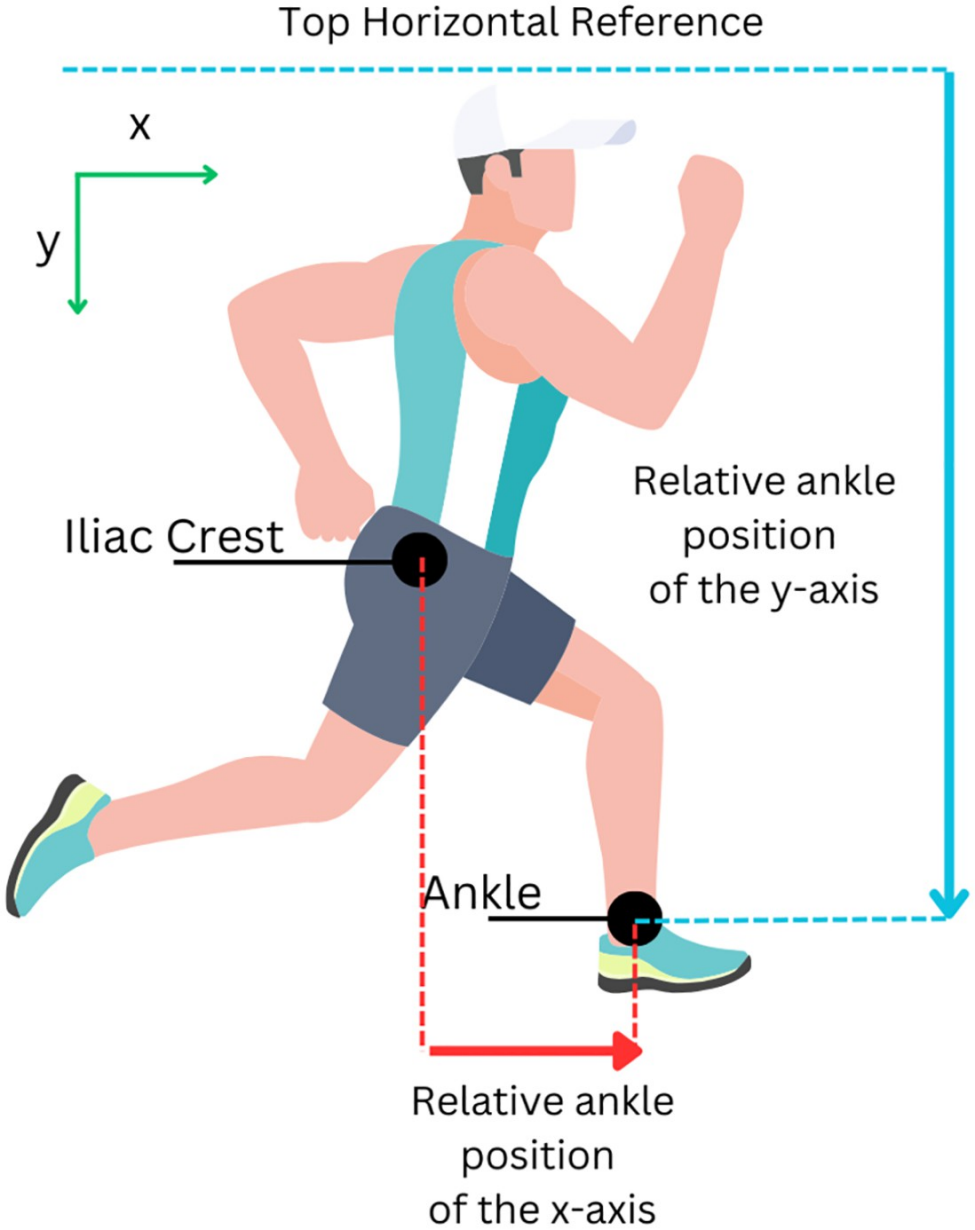
Diagram illustrating relative ankle positions. The relative ankle position in the x-axis represents the ankle’s x-axis position relative to that of the iliac crest. Similarly, the relative ankle position in the y-axis denotes the ankle’s y-axis position relative to the top horizontal reference.

Upon obtaining the relative positions of the x- and y-axes of the human ankle as shown in Fig 2, these relative positions are standardized to have a standard deviation of one and a mean of zero, as calculated by the Eqs 3 and 4.

$$\begin{array}{c} x=\frac{R_{x}-\bar{R_{x}}}{\sigma_{x}} \end{array}$$ (3)

$$\begin{array}{c} y=\frac{R_{y}-\bar{R_{y}}}{\sigma_{y}} \end{array}$$ (4)

*x* and *y* are the standardized relative positions of the x- and y-axes of the human ankle. $\bar{R_{x}}$ and $\bar{R_{y}}$ are the means of the relative positions of the x- and y-axes of the human ankle from the training data. *σ*_*x*_ and *σ*_*y*_ are the standard deviations of the relative positions of the x- and y-axes of the human ankle.

The standardized relative positions of the x- and y-axes of the human ankle are then used as inputs for a neural network.

### Neural network architecture

**Fig 3:**
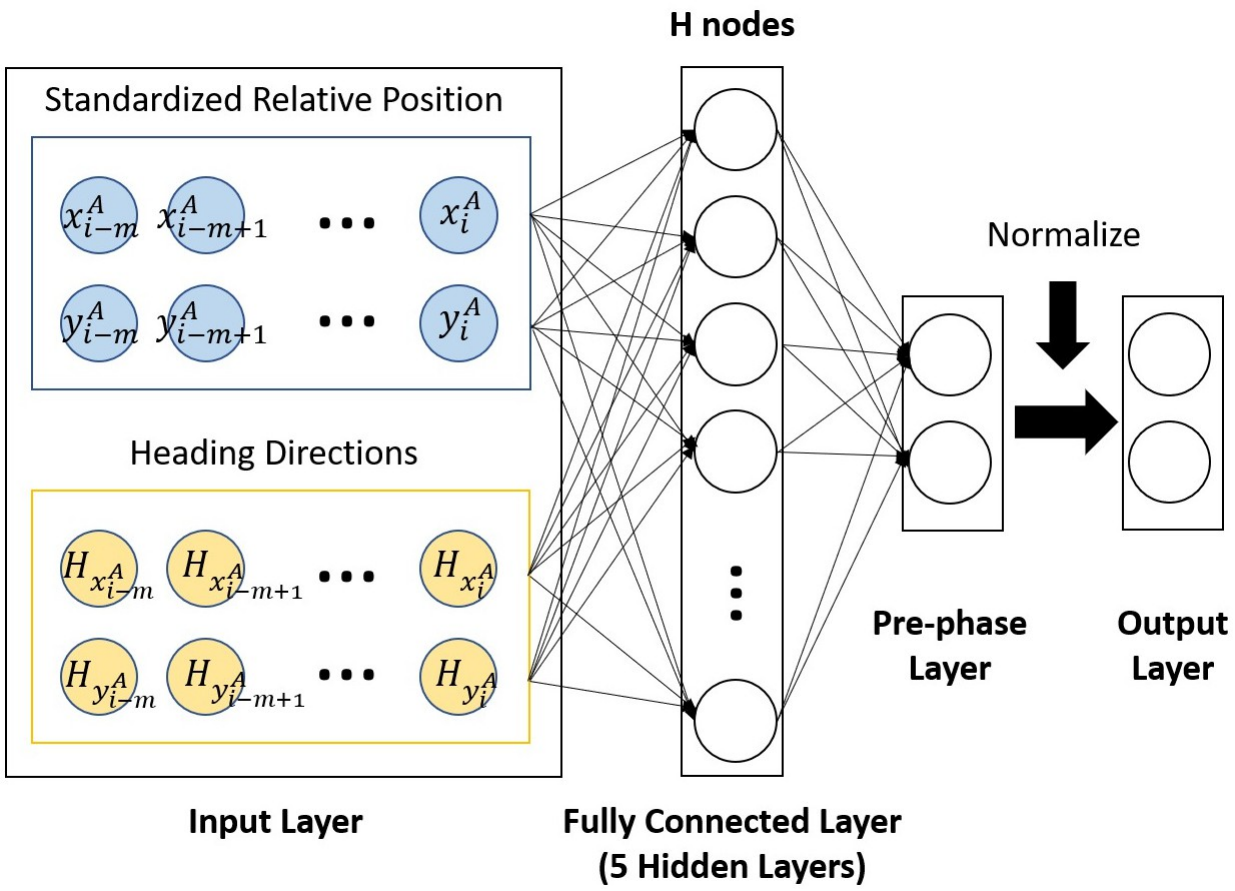
The proposed neural network architecture for left/right lower limb phase learning and extraction. $x_{i-m}^{A},x_{i-m+1}^{A},\ldots ,x_{i}^{A}$ are the standardized relative positions in x-axis at previous and current samples *i* − *m*, *i* − *m* + 1, …, *i* of the left/right ankle position. $y_{i-m}^{A},y_{i-m+1}^{A},\ldots ,y_{i}^{A}$ are the standardized relative positions in y-axis at previous and current samples *i* − *m*, *i* − *m* + 1, …, *i* of the left/right ankle position. $H_{x_{i-m}^{A}},H_{x_{i-m+1}^{A}},\ldots ,H_{x_{i}^{A}}$ are the heading directions of the standardized relative positions in x-axis at previous and current samples *i* − *m*, *i* − *m* + 1, …, *i* of the left/right ankle position. $H_{y_{i-m}^{A}},H_{y_{i-m+1}^{A}},\ldots ,H_{y_{i}^{A}}$ are the heading directions of the standardized relative positions in y-axis at previous and current samples *i* − *m*, *i* − *m* + 1, …, *i* of the left/right ankle position.

The neural network architecture employed in this study bears a resemblance to those utilized in prior studies [19–21]. The input layer comprised four nodes per time sample, including two signal components and two heading direction components, as shown in Fig 3. The heading direction, derived from the slope of a linear regression line fitted to the data points, provides critical information about the directional trend of the data. The architecture incorporated five fully connected hidden layers, with the first four utilizing a hyperbolic tangent activation function and the last connected to the pre-phase layer using a linear activation function. Each of the hidden layers in the architecture comprised 25 nodes. The data from the pre-phase layer, specifically from the two nodes, was normalized to a unit cycle using Euclidean normalization, thereby transforming the information into a phase value at the output layer.

The cost function of this unsupervised neural network consists of four components: the phase progression penalty (PP), the distribution term (D), the singularity penalty (S), and the marginal singularity penalty (M).

The PP enforces a proper phase progression between two consecutive time windows, as described in [43] (Eq 5).

$$\begin{array}{c} PP=\frac{1}{N}\sum_{i=1}^{N}P\left(\text{arctan}\;2\left(\bar{x_{i}}\hat{y_{i}}-\hat{x_{i}}\bar{y_{i}},\bar{x_{i}}\hat{x_{i}}+\bar{y_{i}}\hat{y_{i}}\right)\right) \end{array}$$ (5)

The cross product, represented by the equation $\bar{x_{i}}\hat{y_{i}}-\hat{x_{i}}\bar{y_{i}}$, is equivalent to the sine of an unknown phase progression, denoted as *ν*, where *ν* ∈ (−*π*, *π*] is measured in radians. On the other hand, the dot product, represented by the equation $\bar{x_{i}}\hat{x_{i}}+\bar{y_{i}}\hat{y_{i}}$, is equivalent to the cosine of the unknown phase progression, *ν*. The penalty profile can be calculated by the function P, which was divided into four conditions as defined by the aforementioned reference [43].

$$\begin{array}{c} P\left(\nu \right)=\{\begin{array}{cc} \text{cos}(\frac{\pi }{2}\left(\nu -c\right)/\left(2\pi +c-b\right)) & \text{if}\;\text{-}\pi <\nu \leq c\\ \text{cos}(\frac{\pi }{2}\left(\nu -c\right)/\left(a-c\right)) & \text{if}\;c<\nu \leq a\\ 0 & \text{if}\;a<\nu \leq b\\ \text{cos}(\frac{\pi }{2}\left(\nu -c-2\pi \right)/\left(2\pi +c-b\right)) & \text{if}\;b<\nu \leq \pi \end{array} \end{array}$$ (6)

In the context of left/right lower limb phase learning, the weight of the PP was set to 1. The PP penalty parameters, denoted as *a*, *b*, and *c* in Eq 6, were assigned to $\frac{-2.3\pi }{180}$, $\frac{4\pi }{180}$, and $\frac{-135\pi }{180}$, respectively. The equation used to calculate the PP penalty is presented in Eq 5. The range between *a* and *b* has no phase progression penalty, meaning that two consecutive input windows can naturally progress forward by *b* radians or backward by *a* radians. The indication of *a* and *b* forces all pairs of two consecutive input windows to have a proper phase progression profile. The setting of *b* can be observed by determining the number of frames required to complete one gait cycle. For example, if one complete gait cycle takes 100 frames, the average phase progression of two consecutive input windows will be approximately $\frac{2\pi }{100}$ radians. The setting of *a* in this application started from a negative value because a subject can move in a backward direction in some cases. However, the range between *a* and 0 should be smaller than the range between 0 to *b* to encourage phase movement in an anti-clockwise direction. The setting of *c* should be a value that has a low possibility for phase progression in one input window pair. However, all these parameters need to be fine-tuned until the relative positions of the interested limb painted with the learning phase of the interested limb have a clear and consistent phase progression profile.

The D value in Eq 7 [43], which is an indicator of phase distribution, can be computed by determining the square distance from the center of the unit cycle. The terms presented in the calculation reflect the degree of uniformity with which the phase outputs are distributed on the unit circle. In instances where the phases are not evenly distributed, the centroid will deviate from the center, resulting in a penalty imposed on the neural network.

$$\begin{array}{c} D_{H}=\alpha \left[{\left(\frac{1}{N}\sum_{i=1}^{N}\bar{x_{i}}\right)}^{2}+{\left(\frac{1}{N}\sum_{i=1}^{N}\bar{y_{i}}\right)}^{2}\right] \end{array}$$ (7)

The weight *α* is employed to adjust the relative importance of the distribution penalty term in relation to the phase learning and extraction of the targeted limb(s). The distribution term, represented by *D*_*H*_, pertains to the phase learning and extraction of limb movement in humans.

The singularity penalty in Eq 8 [43], represented by *S*, has been used to prevent the output from the pre-phase layer from approaching the origin. This term can be calculated using the Gaussian function (as represented in Eq 8).

$$\begin{array}{c} S=\frac{\gamma }{N}\left[\sum_{i=1}^{N}\frac{1}{\sigma \sqrt{2\pi }}e^{-\frac{|\vec{V}\left(\Theta ,{\bar{s}}_{i}\right){|}^{2}}{2\sigma^{2}}}\right] \end{array}$$ (8)

While *γ* serves as the weight for the singularity term, it is determined by the relative importance of the singularity penalty. The function $\vec{V}\left(\Theta ,{\bar{s}}_{i}\right)$ returns a 2D vector from the pre-phase layer.

The determination of the weights for each penalty term was achieved through a process of empirical tuning. In the context of this application, the value of the parameter *α* was set to 0.45, and the value of the parameter *γ* was set to 0.55. The cost function, which is computed as the summation of the individual penalty terms, was implemented utilizing TensorFlow. This open-source platform facilitates the automatic differentiation of data flow chains with gradient-based optimizations. TensorFlow has been widely used in the field of machine learning, as outlined in the TensorFlow paper by Abadi et al. [44].

The term M was employed to improve the generalization of phase extraction [43]. Prior to providing data to the neural network, random noise was added to the normal input data. The M can be calculated as follows:

$$\begin{array}{c} M=\frac{\lambda }{N}\left[\sum_{i=1}^{N}\frac{1}{\sigma \sqrt{2\pi }}e^{-\frac{|\vec{V}\left(\Theta ,\xi \left({\bar{s}}_{i},\sigma_{w}\right)\right){|}^{2}}{2\sigma^{2}}}\right] \end{array}$$ (9)

Where $\xi (\bar{s_{i}},\sigma_{w})$ represents a function that adds Gaussian noise with a mean of zero and standard deviation of *σ*_*w*_ to all dimensions of data along with the normalized heading direction data. The weight λ represents the importance of the marginal singularity term. λ was set to 0.55.

### Inferencing step

According to Fig 1, the data obtained from validating all fold’s treadmill walking was utilized to assess time errors (measured in milliseconds). The trained models from each fold were employed to evaluate the testing data in order to ensure that the training neural network model was properly trained. The optimal trained neural network model was identified. Data from overground walking at slow, comfortable, and fast speeds were inputted into the model, which had been trained using treadmill walking data. Additionally, the time errors of the overground walking data, which were not utilized during the training process, were analyzed to determine whether they were significantly different from those of the treadmill walking data from the testing dataset. The overground walking data was collected from 42 subjects [42].

In addition, determining the inferencing time is important to understand the possibility of real-time implementation. The computational time during executing the inferencing step was also observed. The processing time to obtain the gait phase from the pre-trained model is approximately 17.33 milliseconds, based on our hardware configuration, which includes a 12th Gen Intel(R) Core(TM) i7-12700KF processor operating at 3.60 GHz and 32 GB of installed RAM. This demonstrates that our algorithm possesses sufficient capability for real-time application.

### Verification the performance of pre-trained neural network model

**Fig 4:**
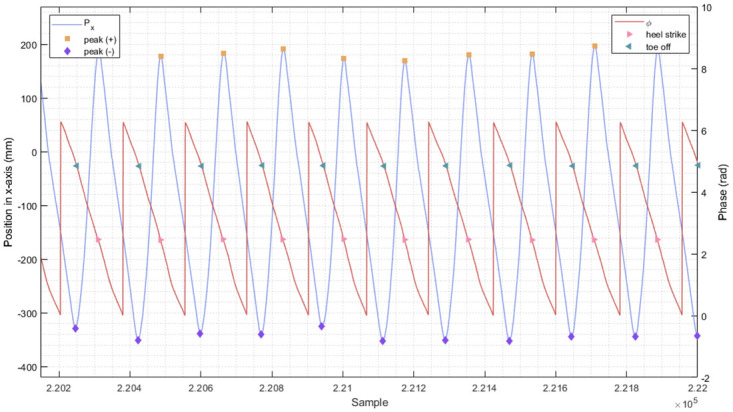
Dynamic representation of position and phase over sample points. The blue line depicts the relationship between the sample and position (measured in millimeters) on the x-axis during treadmill walking data. The yellow squares represent positive peaks, which correspond to heel strike events, and the purple diamonds signify negative peaks, which indicate toe-off events. The heel strike and toe-off events serve as the ground truth for foot contact. In the context of our gait analysis, heel strike events are defined as the point at which the horizontal velocity of the foot transitions from positive to negative, while toe-off events are marked by the transition from negative to positive. This approach is consistent with the method employed by Zeni et al. for detecting heel strike and toe-off events [45]. for detecting heel strike and toe-off events. In addition, the red line illustrates the correlation between the sample and phase (represented in radians) during the same treadmill walking data. The green left-pointing triangles denote phase values during toe-off events, while the pink right-pointing triangles depict phase values during heel strike events.

To assess the effectiveness of our pre-trained neural network model, we used the time error metric, expressed in milliseconds, to evaluate its performance. The error serves as an indicator of the temporal detection disparity between the goal phase and output phase of the proposed algorithm. First, the standard walking events of heel strike and toe-off were identified through the detection of positive and negative peaks of the position in x-axis in the graph. These peaks correspond to abrupt changes in direction at the heel strike and toe-off gait events, respectively, as illustrated in Fig 4. Our proposed method recognized these phases by utilizing constant phase values established at heel strike and toe-off events. Next, the average event values for heel strike and toe-off were calculated using phase values from our pre-trained model, which were trained on training data collected at these events. These events were identified through the detection of positive and negative peaks of the position in x-axis, as shown in Fig 4.

The comparison of time errors (ms) in detecting left/right heel strike and left/right toe-off events during treadmill walking across different folds was conducted using one-way ANOVA with the Bonferroni method to evaluate the error-balancing strategy performance of our neural network model. Specifically, the time errors in detecting left/right heel strike and left/right toe-off gait events were compared across four folds, encompassing the total gait cycles from the treadmill walking data. Significant differences between the four folds with respect to these gait events would suggest that the neural network employs varied strategies to minimize error, potentially balancing errors across different sub-gait events. These variations in pre-trained models result in differing time errors for specific gait events. Consequently, by analyzing the time errors associated with detecting these gait events, one can identify the model best suited for a particular application. For instance, if an application necessitates prioritizing the detection of left toe-off gait events, the pre-trained model exhibiting the lowest time delays for left toe-off events should be selected.

The statistical differences in time errors for detecting walking events under various conditions—namely, treadmill walking and overground locomotion at slow, comfortable, and high speeds—were analyzed using one-way ANOVA with the Tukey-Kramer post-hoc test to evaluate the adaptability of the pre-trained model to unseen data from different settings. This analysis encompassed treadmill walking and overground locomotion at varying speeds. The average time errors for detecting left/right heel strike and left/right toe-off gait events across all folds from all gait cycles were statistically compared. The objective was to determine whether the model, trained on treadmill data at eight distinct speeds, could generalize to overground locomotion at three different speeds. Significant differences between these conditions would suggest that treadmill walking speed may be a parameter influencing the time errors in detecting gait events, as the time errors between different speeds of overground locomotion were found to be significantly different. Consequently, selecting a specific treadmill walking speed could be a potential solution to decrease time errors in detecting gait phases during specific overground locomotion.

### Comparing pre-trained models trained on individual treadmill speeds to those trained on combined treadmill data across all speeds

To investigate the influence of treadmill training data speeds on performance with overground locomotion testing data at varying speeds, we evaluated testing data at slow, comfortable, and high speeds against optimal pre-trained models derived from treadmill walking data categorized into three speed sets. The slow speed set includes speeds at 40%, 55%, 70%, and 85% of self-selected dimensionless speed. The comfortable speed set comprises data at 100% of self-selected dimensionless speed. The fast speed set encompasses speeds at 115%, 130%, and 145% of self-selected dimensionless speed. Subsequently, we compared the time errors resulting from the inference process of pre-trained models using specific speeds with those of models trained on combined speeds to examine the impact of treadmill walking speeds on the time errors of untrained overground walking data across three different speeds.

The methodology employed for hypothesis validation encompassed the development of specific pre-trained models tailored to distinct treadmill speeds: slow, comfortable, and high. Each speed category comprised individual models trained using four cross-validation folds, as depicted in Fig 1. Subsequently, the average time errors of the testing data from overground locomotion at various speeds, which were derived from inferences made by a pre-trained model based on specific treadmill walking speeds, were computed. This process aimed to identify the optimal pre-trained model from the four folds that best exemplifies the neural network approach for each speed category.

In addition to the specific speed models, a composite model encompassing training across all treadmill speeds was selected based on superior performance across the four folds. This composite model served as a representative training approach across a range of treadmill speeds. Subsequently, this selected composite model was employed to predict time errors in detecting gait events from overground locomotion testing data at slow, comfortable, and high speeds. The ensuing analysis compared the time errors obtained using the specific pre-trained models with those derived from the composite model trained across all speeds. This comparative assessment aimed to elucidate the impact of varying treadmill walking speeds on the efficacy of pre-trained models in predicting gait event detection errors during overground locomotion at different speeds. Statistical analysis was conducted using the T-test method to ascertain significant differences between the model performances.

## Results and discussions

### Phase consistency

**Fig 5:**
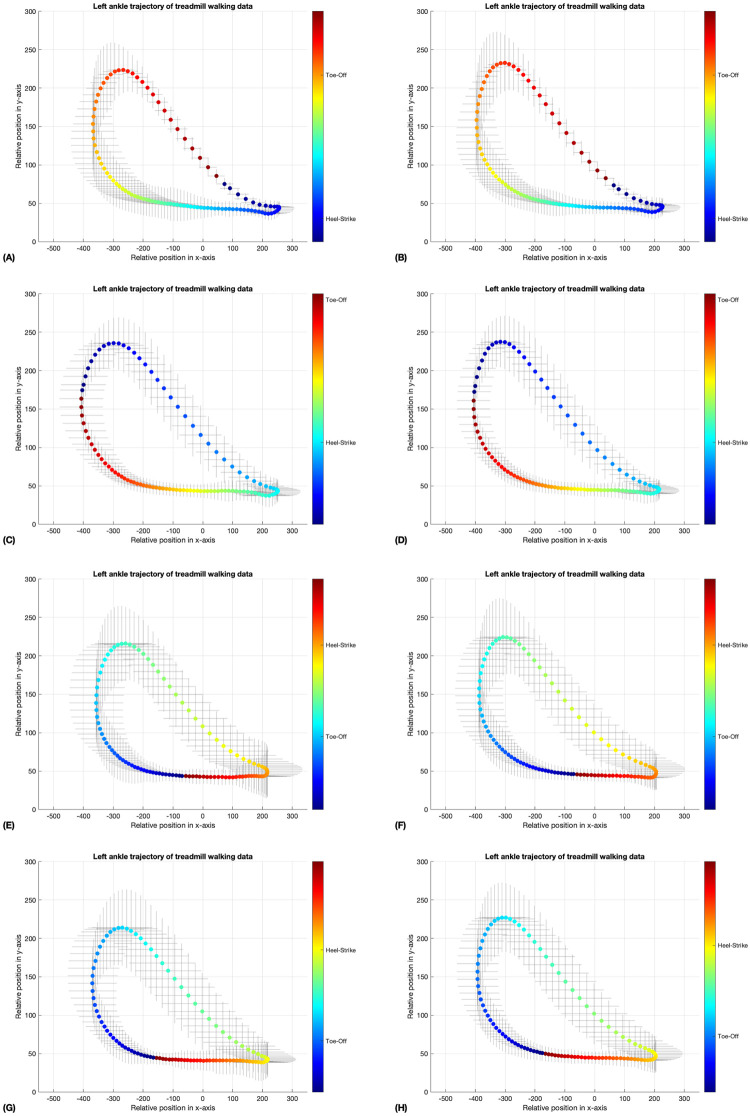
The average trajectory of the left ankle, labeled by phase during treadmill walking, was examined using data obtained from both the validation datasets (A, C, E, G) and the testing datasets (B, D, F, H). The error bar surrounding the mean trajectory represents the standard deviation at each point along the trajectory. The phase of each trial was represented by a colorbar in radians, with the number of rows representing the fold number (1 to 4). (A, B) The heel strike phase is represented by the color blue, while the toe-off phase is indicated by the color orange. (C, D) The heel strike phase is represented by the color cyan, while the toe-off phase is indicated by the color red. (E, F) The heel strike phase is represented by the color orange, while the toe-off phase is indicated by the color blue. (G, H) The heel strike phase is represented by the color yellow, while the toe-off phase is indicated by the color blue.

**Fig 6:**
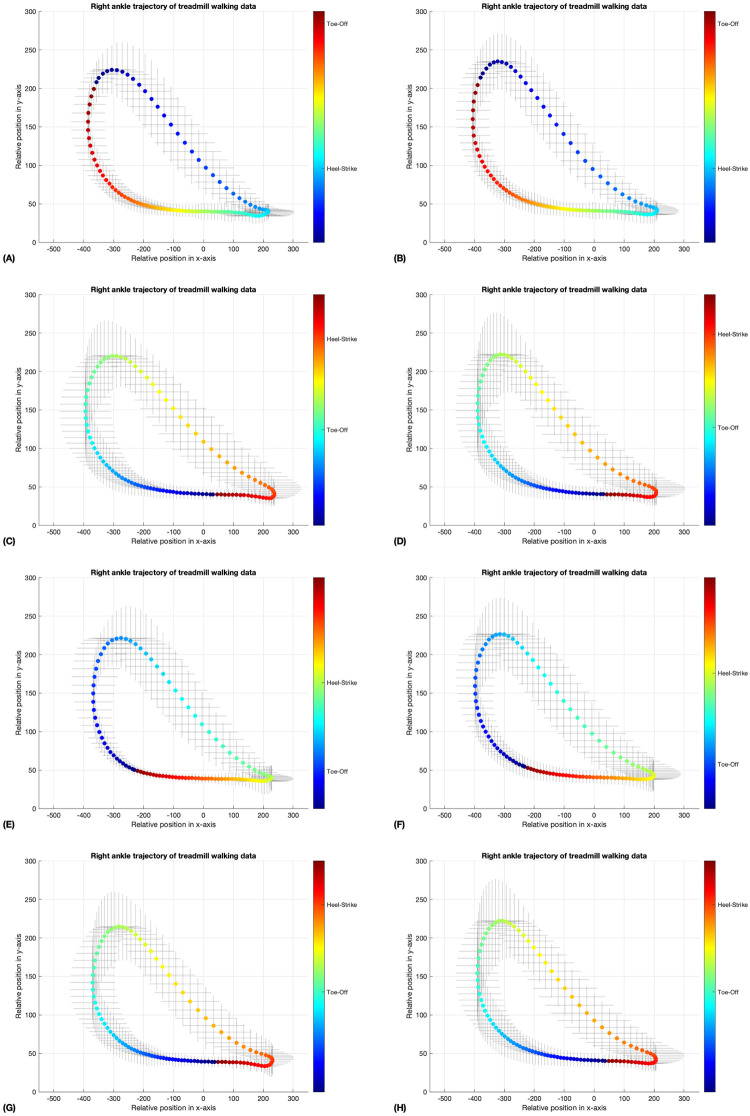
The average trajectory of the right ankle, labeled by phase during treadmill walking, was examined using data obtained from both the validation datasets (A, C, E, G) and the testing datasets (B, D, F, H). The error bar surrounding the mean trajectory represents the standard deviation at each point along the trajectory. The phase of each trial was represented by a colorbar in radians, with the number of rows representing the fold number (1 to 4). (A, B) The heel strike phase is represented by the color blue, while the toe-off phase is indicated by the color red. (C, D) The heel strike phase is represented by the color orange-red, while the toe-off phase is indicated by the color turquoise. (E, F) The heel strike phase is represented by the color green, while the toe-off phase is indicated by the color blue. (G, H) The heel strike phase is represented by the color orange-red, while the toe-off phase is indicated by the color turquoise.

**Fig 7:**
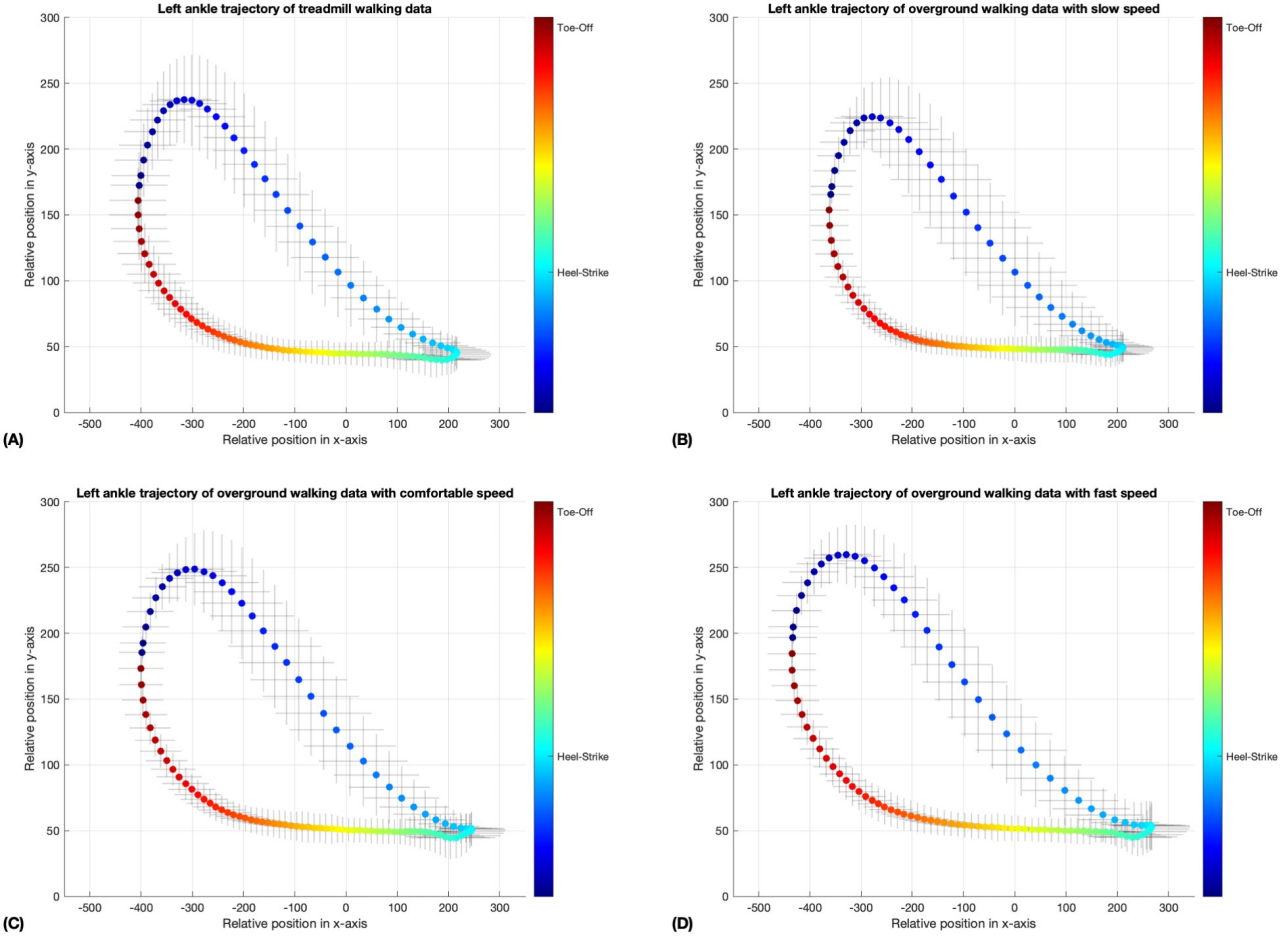
The average trajectory of the left ankle, annotated with phase information, was assessed under both treadmill and overground walking conditions, with speed variations ranging from slow to fast. The error bar surrounding the mean trajectory indicates the standard deviation at each point along the trajectory. The data are presented in four segments: (A) illustrates the results for treadmill walking, (B) for slow-speed overground walking, (C) for comfortable-speed overground walking, and (D) for fast-speed overground walking. The phase calculated by neural network model from fold 2 of each trial was depicted by the colorbar, displayed in radians. (A, B, C, D) The heel strike phase is represented by the color cyan, while the toe-off phase is indicated by the color red.

**Fig 8:**
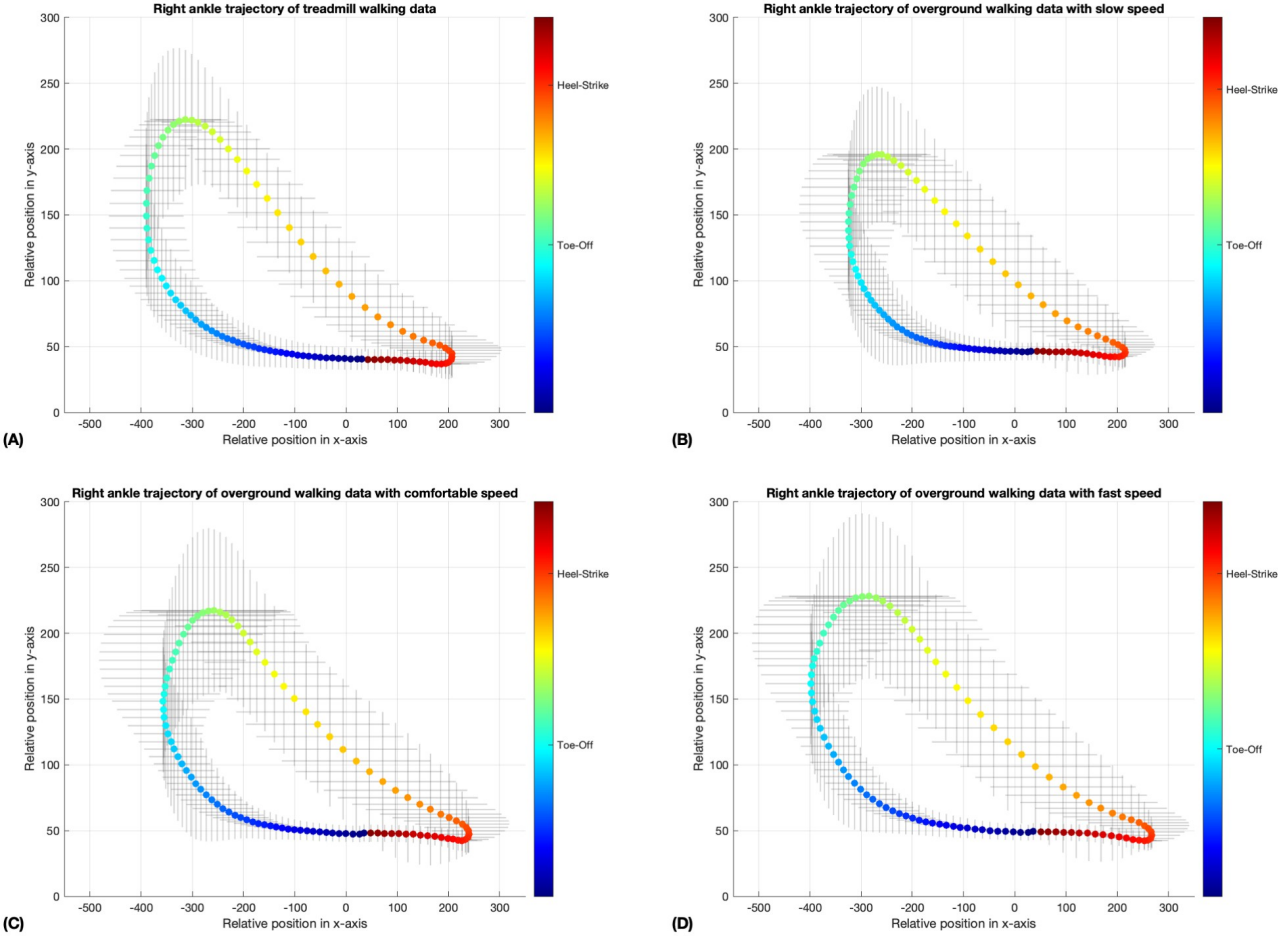
The average trajectory of the right ankle, annotated with phase information, was assessed under both treadmill and overground walking conditions, with speed variations ranging from slow to fast. The error bar surrounding the mean trajectory indicates the standard deviation at each point along the trajectory. The data are presented in four segments: (A) illustrates the results for treadmill walking, (B) for slow-speed overground walking, (C) for comfortable-speed overground walking, and (D) for fast-speed overground walking. The phase calculated by neural network model from fold 2 of each trial was depicted by the colorbar, displayed in radians. (A, B, C, D) The heel strike phase is represented by the color orange-red, while the toe-off phase is indicated by the color turquoise.

In Figs 5–8, the coloring scheme represents the different phases of gait, with the color yellow in Fig 7B–7D indicating the mid-swing phase of the left ankle, for instance. These results were generated by a neural network model that underwent 10,000 iterations of training through unsupervised learning on an unlabeled dataset. This method may lead to variations in the initial gait phases for the walking data in the four-folds, as shown in Figs 5 and 6. Nevertheless, the standard deviation error bars depicted in Figs 5–8 indicate that, within similar phases, the deviations in x- and y-positions from the average trajectory are minimal. This suggests that our phase extraction method maintains consistency across all phases.

### Quantitative results

#### The performance of pre-trained neural network model

**Fig 9:**
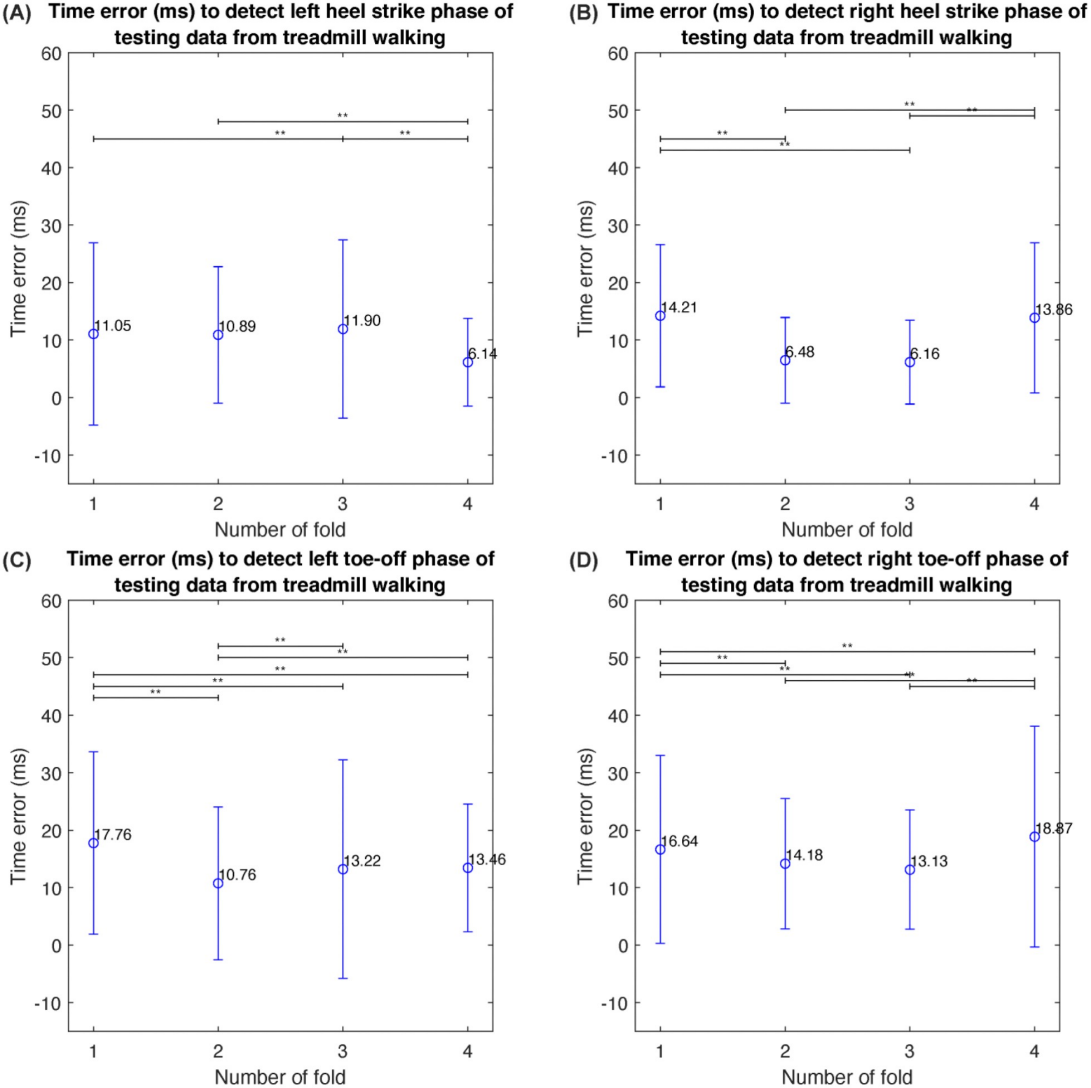
The time error in milliseconds required to detect the (A) left heel strike event, (B) right heel strike event, (C) left toe-off event, and (D) right toe-off event, as determined by the neural network model of each fold, using testing data is reported. Significance levels are indicated by an asterisk (*) with a p-value of less than 0.05 and a double asterisk (**) with a p-value of less than 0.01.

A 4-fold cross-validation method was employed to evaluate the proposed neural network models in detecting gait phases in terms of the average time error. As depicted in Fig 9, the results reveal variations in time errors across different folds. These results demonstrate that each pre-trained model employs a distinct strategy to balance the time error associated with each gait event, as evidenced by the significant variations in time error for detecting gait events across different folds. Consequently, if an application necessitates precise detection of specific gait events, it is essential to evaluate the time error associated with gait event detection. This evaluation will help determine which pre-trained model is most suitable for the application’s requirements. A summary of the average time errors for all four gait phases across all folds is presented in Table 2. The average time errors for the testing and validation data from all folds are 11.96±12.74 ms, which are sufficient for detecting continuous gait phases in real-time applications [46–50].

**Table 2:** Gait phase detection using time error analysis in treadmill walking data.

	Time Error (ms)							
	Left Trajectory				Right Trajectory			
	Toe-off Event		Heel Strike Event		Toe-off Event		Heel Strike Event	
	Average	SD	Average	SD	Average	SD	Average	SD
Validation data								
Fold1	14.85	11.39	11.81	15.25	9.39	14.14	10.06	16.46
Fold2	10.62	12.83	15.26	16.22	11.11	6.60	9.66	7.24
Fold3	12.55	13.11	11.13	16.07	8.90	6.25	7.41	8.90
Fold4	11.76	9.38	8.15	10.53	14.59	16.78	16.46	18.95
Average	12.45	11.68	11.59	14.52	11.00	10.94	10.90	12.89
Testing data								
Fold1	17.76	15.87	11.06	15.83	16.65	16.34	14.21	12.36
Fold2	10.76	13.31	10.89	11.85	14.18	11.33	6.48	7.43
Fold3	13.22	19.04	11.90	15.48	13.13	10.38	6.16	7.30
Fold4	13.46	11.11	6.14	7.62	18.87	19.22	13.86	13.03
Average	13.80	14.83	10.00	12.70	15.71	14.32	10.18	10.03

**Fig 10:**
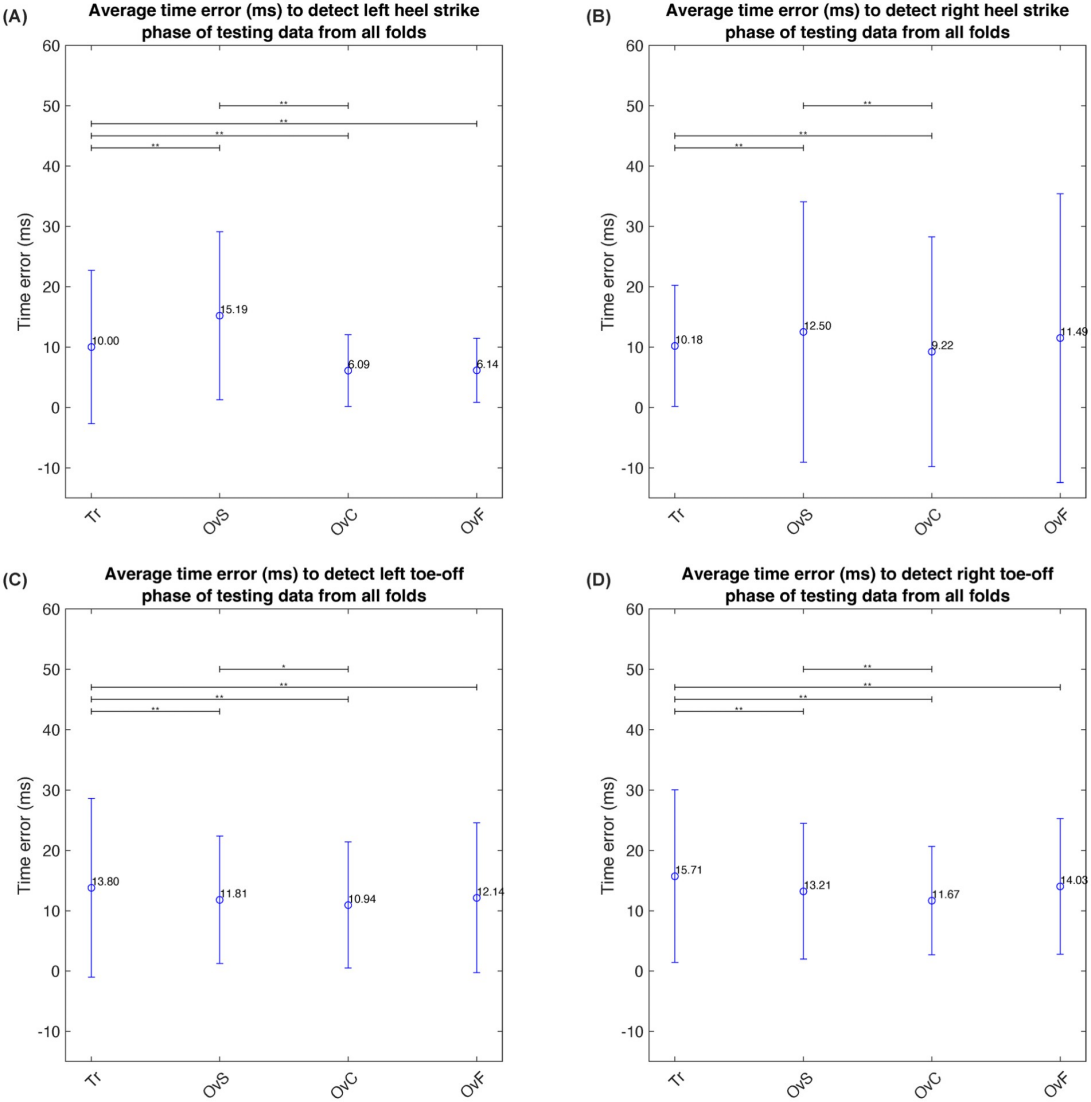
The average time error in milliseconds for detecting the (A) left heel strike, (B) right heel strike, (C) left toe-off, and (D) right toe-off events across all folds. Tr represents treadmill walking, while OvS, OvC, and OvF denote overground locomotion at slow, comfortable, and fast speeds, respectively. Statistical significance was determined by an asterisk (*) denoting p-values less than 0.05, and a double asterisk (**) denoting p-values less than 0.01.

To evaluate the effectiveness of the pre-trained neural network model developed based on treadmill walking data on untrained or overground walking data, we fed the untrained treadmill walking data or overground walking data into the pre-trained model. Our findings indicate substantial disparities among the walking conditions, as illustrated in Fig 10, which presents the mean temporal inaccuracies derived from all folds of the pre-trained model. Notably, the average time errors associated with detecting the left toe-off event and right toe-off event of the overground walking condition at all speeds were significantly lower than those of the treadmill walking condition, as evidenced by Table 3. This observation suggests that the pre-trained neural network model is capable of proficiently operating under diverse walking conditions beyond the training data, which may be attributed to the inclusion of treadmill walking data gathered at varying speeds. When comparing our findings with traditional studies [51], it becomes evident that our proposed neural network-based method offers enhanced flexibility and necessitates minimal setup time. Instruments such as plantar pressure insoles, force sensors, and IMU sensors are commonly utilized for detecting gait events like heel strike and toe-off. While these tools, particularly plantar pressure insoles, are more cost-effective than the motion capture system employed in our study, they may offer increased accuracy for specific gait event detection. However, our method distinguishes itself by detecting a continuous gait phase, making it especially suitable for real-time robotic control.

**Table 3:** Gait phase detection using time error analysis in treadmill walking and overground.

Walking condition	Average time error (ms) from all folds							
	Left Trajectory				Right Trajectory			
	Toe-off Event		Heel Strike Event		Toe-off Event		Heel Strike Event	
	Average	SD	Average	SD	Average	SD	Average	SD
Treadmill walking data	13.80	14.83	10.00	12.69	15.71	14.32	10.18	10.03
Overground locomotion with slow speed	11.81	10.57	15.19	13.90	13.21	11.25	12.50	21.59
Overground locomotion with comfortable speed	10.94	10.48	6.09	5.92	11.67	8.96	9.22	19.02
Overground locomotion with fast speed	12.14	12.41	6.14	5.29	14.03	11.23	11.49	23.92
Average	12.17	12.07	9.36	9.45	13.66	11.44	10.85	18.64

Regarding the detection of left and right heel strike events, our results indicated that the average time errors during overground locomotion at slow speeds were significantly higher than those observed during treadmill walking and overground locomotion at a comfortable speed. While the average time errors during overground locomotion at slow speeds were higher than those during overground locomotion at high speeds, this difference was not found to be statistically significant. We attribute these outcomes to the inclusion of gait-speed treadmill walking conditions, which cover a broader range of fast speeds (145% of the self-selected dimensionless speed) in comparison to slow speeds (40% of the self-selected dimensionless speed). It is noteworthy that the mean temporal errors associated with detecting the left/right heel strike and left/right toe-off events from overground walking data at a comfortable speed were the lowest relative to the other conditions. This finding may be explained by the fact that the participants naturally walked at their own comfortable speed, leading to data that approximates the training data and, consequently, the lowest average time error. The left/right toe-off event detection results (refer to Fig 10C and 10D) indicated that the average time errors associated with the treadmill walking condition were the highest, followed by those of overground locomotion at high speeds, overground locomotion at slow speeds, and overground locomotion at comfortable speeds. The lower time errors observed in the detection of the left/right toe-off events during overground locomotion at a slow speed as compared to that at a high speed can be attributed to the relative ease of detecting sudden changes at slower speeds. This is due to the fact that slower movements allow for more distinct changes in gait [52], which are easier to detect than at higher speeds, where changes may be more subtle and difficult to discern. However, our analysis did not detect any significant difference in the mean temporal error across all detected gait phases (including left/right heel strike and left/right toe-off events) during overground locomotion at both slow and high speeds. The substantial variations in time errors associated with detecting gait events under different walking conditions indicate that the walking speed of the training data may influence the accuracy of gait event detection. Consequently, a pre-trained model tailored to individual treadmill speeds was developed to evaluate whether this approach reduces time errors in gait event detection compared to a model trained on combined treadmill data across all speeds. The results of this comparative analysis are detailed in the next subsection.

In general, although there were some variations in the time errors, the average time errors for detecting gait events (left/right toe-off and left/right heel strike) from the testing data across all walking conditions were found to be 11.51 ± 12.90 ms (refer to Table 3). This accuracy is deemed sufficient for real-time applications requiring the detection of continuous gait phases [46–50]. Notably, the average time errors from overground walking at different speeds were 11.20 ± 12.88 ms, which is lower than that of treadmill walking data. This outcome indicates that the transfer learning model is effective for this application. Thus, a straightforward setup involving cameras and a treadmill can be utilized to capture walking signals to acquire a pre-trained model. Once a highly performing pre-trained model is obtained, it can be applied to overground locomotion data. However, gathering such data presents challenges due to the need for expansive areas and expensive motion capture systems. Additionally, the use of cameras introduces complications for real-time applications, primarily due to occlusion issues. To address this challenge, we propose leveraging positional data from commercially available IMU systems. For example, Xsens sensors can utilize 3D joint angles and segment positions to provide precise measurements of body accelerations and positions [53]. Integrating our pre-trained model with these systems enables continuous gait phase detection, thereby mitigating the constraints associated with marker-based methods.

#### Comparison of time errors to detect gait events between inferencing results from pre-trained model trained on individual treadmill speeds to those combined treadmill data across all speeds

Table 4 demonstrates that the time errors in detecting gait events using a model trained on specific treadmill speeds (slow, comfortable, and fast) are significantly lower than those obtained using a model trained on combined treadmill data across all speeds. This highlights the advantage of using speed-specific training data, which results in better performance in detecting gait events at different walking speeds. Thus, for applications requiring accurate speed-related performance, pre-trained models with data from specific speed ranges are recommended.

**Table 4:** Comparison of time errors in detecting gait events using models trained on individual versus combined treadmill speeds.

Speed of overground walking data	Average time error (ms) to detect gait events from overground walking data ± SD		Significant difference
	Pre-trained model on individual treadmill speeds	Pre-trained model on combined treadmill data across all speeds	
Slow	8.64 ± 16.42	11.47 ± 14.48	**
Comfortable	7.23 ± 9.63	8.87 ± 11.81	**
Fast	8.28 ± 16.33	10.01 ± 15.75	**

Statistical significance was determined by a double asterisk (**) denoting p-values less than 0.01.

A multitude of studies have developed algorithms aimed at detecting foot contact with minimal timing errors, each employing different methodologies and focusing on various populations. For example, Romijnders et al. employed a deep learning approach to identify gait events, achieving a median timing error close to zero, with the maximum median error being -5 ms [54]. Their analysis included 157 participants across seven distinct groups: young adults, older adults, individuals with Parkinson’s disease, recent stroke survivors with symptoms, individuals with multiple sclerosis, those with chronic low back pain, and participants with either undiagnosed conditions or diagnoses not fitting within these categories. The study utilized an IMU sensor with a sampling rate of 200 Hz during overground walking at three different speeds: normal walking speed, fast speed, and half of the normal walking speed.

Another study by Mo and Chow developed three algorithms—S-method, M-method, and MS-method—for detecting gait events during overground running using IMU sensors [55]. The S-method exhibited the lowest time error in detecting initial contact at 4.2 ms, while the M-method recorded the lowest time error in detecting final contact at 8.8 ms. In contrast, Aminian et al. utilized wavelet transformation to detect initial contact events, reporting timing errors between 7 and 13 ms with 95% confidence [56]. For final contact events, the timing errors ranged from -5 ms to 4 ms, also within a 95% confidence interval. These findings were observed in both young and older adults under treadmill and overground walking conditions. Additionally, another study focused on child subjects during overground walking reported timing errors ranging from -16 ms to 1 ms for initial contact and from 37 ms to 63 ms for final contact, also within a 95% confidence interval [57].

Timing accuracy was further examined by Kim and Lee from Korea, who developed an IMU-based algorithm for detecting foot-ground contact [58]. Their study, involving 10 adult participants with no history of musculoskeletal disorders, found a time delay of 60 ms in detecting final contact during stair ascent. Similarly, another study measured timing errors in detecting initial and final contact across four groups of subjects: healthy elderly individuals, those with hemiparesis, Parkinson’s disease, and choreic movement disorders [59]. The maximum mean timing error observed was 11 ms during comfortable speed and 22 ms at a faster speed.

Maqbool et al. focused on detecting foot-flat start and heel-off events using an acceleration-based algorithm, employing proprietary rules [60]. This method was tested on both a transfemoral amputee and healthy subjects. For healthy subjects, the mean timing error in detecting initial contact was 17 ms, while the error for detecting final contact was -16 ms. For transfemoral amputees, the mean timing error in detecting initial contact at the intact limb was 12 ms, with the final contact error being -24 ms.

While these studies demonstrate that several algorithms are capable of detecting foot contact with varying levels of time error using data from IMU sensors, none have successfully achieved continuous detection of gait phases—a critical requirement for real-time exoskeleton control. Our proposed algorithm seeks to address this gap. Although its time error may not represent the highest level of performance, it exhibits significant potential for integration with IMU-based motion capture systems, meriting further investigation. The ability to achieve continuous gait phase detection could significantly advance the development of real-time exoskeletons, potentially improving their responsiveness and effectiveness in rehabilitation settings. However, the current algorithm’s performance under varied conditions and with different populations requires further testing. Future research could explore optimizing its timing accuracy and expanding its applicability to other locomotor tasks.

## Conclusion

In this study, we advanced our previous work on continuous gait phase detection using neural networks. Previously, our approach exclusively used treadmill data for both training and testing. The current study introduces an adaptation of this pre-trained treadmill data model for overground walking data, highlighting the application of transfer learning in biomechanics. Our results indicate a discrepancy in time error between treadmill and overground walking data, attributed to differences in walking conditions. However, the average time error below 12.42 ms for both conditions emphasizes the neural network’s efficiency in real-time applications across these conditions. To achieve reduced average time errors in detecting gait events during overground locomotion, it is imperative to train the model using data that closely corresponds to the speed range of the testing dataset. Specifically, the average time error from models tailored to specific speeds (8.05 ms) was significantly lower than that from models trained on combined speed data (10.12 ms). Future research will focus on integrating our proposed algorithm into an IMU-based motion capture system, with the aim of evaluating its performance against existing real-time algorithms for IMU-based contact detection. Additionally, we plan to explore the use of unsupervised neural networks to extract arm phases and synchronize it with lower limb phases. Achieving this could lead to improved control of lower limb exoskeletons and potentially accelerate the recovery of neural pathways between thoracic and lumbar central pattern generators.
